# Enhancing the efficacy of *Hypericum perforatum* in the treatment of an experimental model of multiple sclerosis using gold nanoparticles: an *in vivo* study

**DOI:** 10.22038/AJP.2022.19574

**Published:** 2022

**Authors:** Mahmoud Mahmoudi, Maryam Rastin, Mohammad Kazemi Arababadi, Akbar Anaeigoudari, Reza Nosratabadi

**Affiliations:** 1 *Immunology Research Center, Department of Immunology and Allergy, School of Medicine, Mashhad University of Medical Sciences, Mashhad, Iran*; 2 *Department of Laboratory Sciences, Faculty of Paramedicine, Rafsanjan University of Medical Sciences, Rafsanjan, Iran*; 3 *Department of Physiology, School of Medicine, Jiroft University of Medical Sciences, Jiroft, Iran*; 4 *Department of Medical Immunology, Afzalipour Faculty of Medicine, Kerman University of Medical Sciences, Kerman, Iran*; 5 *Immunology of Infectious Diseases Research Center, Research Institute of Basic Medical Sciences, Rafsanjan University of Medical Sciences, Rafsanjan, Iran*

**Keywords:** Hypericum perforatum L. Multiple sclerosis, Experimental autoimmune, encephalomyelitis, Gold nanoparticle, Myelin oligodendrocyte glycoprotein

## Abstract

**Objective:**

*Hypericum perforatum* is a herbal medicine used in traditional medicine for the treatment of depression due to its antidepressant and anti-inflammatory activities. Therefore, we evaluated the therapeutic efficacy of *H. perforatum* extract (HPE) in combination with gold nanoparticles (HPE-GNP) against experimental autoimmune encephalomyelitis (EAE), an animal model of multiple sclerosis.

**Materials and Methods:**

EAE was induced in C57BL/6 mice with subcutaneous injection of MOG35-55 emulsified in complete Freund's adjuvant, and intraperitoneal pertussis toxin. Mice were treated with drugs in free (HPE) and nano-form (HPE-GNP) preparations. Splenocytes were isolated from all mice and the level of inflammatory and anti-inflammatory cytokines were evaluated by ELISA. The expression of T cells' transcription factors was also assessed using Real-Time PCR.

**Results:**

Clinical score was reduced after HPE-GNP treatment. This change was associated with a decrease in the incidence and infiltration of inflammatory cells into the central nervous system. Additionally, treatment with HPE-GNP decreased the level of pro-inflammatory cytokines (IFN-γ, IL-17A and IL-6) and increased anti-inflammatory cytokines (TGF-β, IL-10 and IL-4). The real-time analysis revealed a decrease in the level of *T-bet* and *ROR-γt* but an increase in *FoxP3* and *GATA3* expression.

**Conclusion:**

The current study demonstrated that HPE-GNP could potentially reduce clinical and pathological complications of EAE, but laboratory data showed that HPE-GNP was significantly more effective than HPE in the treatment of EAE.

## Introduction

Multiple sclerosis (MS) is an immune-mediated and neurodegenerative disease of the central nervous system (CNS) and it is the most common cause of neurological disability diagnosed in young adults (Lassmann and Bradl, 2017 ▶). The etiology of this complex disease is poorly understood and there is no curative therapy for MS (Dobson and Giovannoni, 2019 ▶). Experimental autoimmune encephalomyelitis (EAE) is a useful animal model of MS in which, CNS inflammation and demyelination have been observed to be similar to that of MS (Procaccini et al., 2015 ▶).

It is known that myelin-specific Th1 cells play an essential role in the pathogenesis of EAE/MS (Martin et al., 2016 ▶). However, IFN-γ-knocked out mice (the key cytokine in Th1 differentiation) develop more severe EAE, suggesting that Th1 cells may not be the only immune cells involved in the disease (Lovett-Racke et al., 2011 ▶). Some investigations have established the central role of Th17 in EAE pathogenesis (McGinley et al., 2018 ▶). Th17 cells are characterized by expression of transcription factor ROR-γt and IL-17 production (Capone and Volpe, 2020 ▶). Inhibition of Th17 cells or IL-17 cytokine leads to amelioration of EAE, whereas adaptive transfer of them is associated with the disease aggravation (Acharya et al., 2018 ▶).

On the other hand, regulatory T cells (Treg cells) and Th2 play a protective role in EAE/MS progression (Danikowski et al., 2017 ▶). Several investigations have demonstrated that development and function of Th1 cells are suppressed by Th2-mediated mechanisms (Bretscher, 2019 ▶). Therefore, Th2 and its related cytokines (such as IL-4) may have a protective role in EAE/MS. 

Although a wide range of research has been done for EAE/MS, treatment of MS remains unknown. On the other hand, current medications have side effects and minor efficacy in patients who suffer from MS (Gajofatto and Benedetti, 2015 ▶).

Herbal remedies are the most promising agents and several studies have indicated that the use of herbal remedies has growing interest in the treatment of infectious and non-infectious diseases due to their low adverse effect and toxicity (Sriwijitalai and Wiwanitkit, 2020 ▶). 

St. John’s wort or *Hypericum perforatum* L. (HP) is a herb of the Hypericaceae family (Dauncey et al., 2019 ▶). In Chinese and Iranian traditional medicine, it has been used for treatment of mild to moderate depression (Dauncey et al., 2019 ▶). Today, several investigations use HP in the treatment of neurodegenerative diseases such as Alzheimer's and Parkinson's disease (Gomez et al., 2013 ▶; Kraus et al., 2007b ▶; Vecchia et al., 2015 ▶). Altan et al. has also indicated that HP plays a role as an antioxidant, anti-inflammatory and wound-healing herbal medicine (Altan et al., 2018 ▶).

Nowadays, to overcome some limitations and side effects of current medications, novel therapeutic strategies such as nanotechnology have been expanded in medicine. 

Gold nanoparticles (GNPs) are a good candidate because of their potential in binding to different types of molecules as they have ability in drug delivery and drug release, as well as their low toxicity (Nosratabadi et al., 2016a ▶). 

Recently, green synthesis of GNPs by plant extracts has gained great interest due to the plants being inexpensive and nontoxic (Teimuri-Mofrad et al., 2017 ▶).

In this study, we propose a new form of *H. perforatum* extract (HPE) bound to the GNPs (HPE-GNP) to evaluate the effects of HPE in nano form in the treatment of EAE. 

## Materials and Methods

**Plant extract preparation**

The aerial parts of HP were collected in spring from mountain hills of Baft and confirmed in Herbarium of Department of Biology, Shahid Bahonar University of Kerman (*Hypericum perforatum* number:3837, MIR herbarium). 

The plant materials were dried at room temperature in the shade and then, ground into fine powder. Then, 100 g of the powder was used for hydro-alcoholic extraction by ethanol-water. The mixture was placed on a shaker for 72 hr and the obtained extract was filtered three times. The filtrates were placed at 40^O^C under vacuum for complete removal of solvents. After extract preparation, three concentrations were made from the stock and were expressed as milligrams of dried extract per kilogram of mouse body weight. Endotoxin content in the extracts was determined using a limulus amoebocyte lysate assay. Less than 0.01 U/µg endotoxin was present in the extract samples.

**Gold nanoparticles (GNPs) synthesis**

Nanoparticles were synthesized by reduction of tetrachloroauric acid (HAuCL4) by sodium citrate. Briefly, 10 mg of tetrachloroauric acid (Sigma Aldrich, St. Louis, MO, USA) was dissolved in 25 ml of deionized water. This resulted in a 1 mM HAuCl_4_ solution. Then, the solution was heated on a hot plate with a magnetic stirrer. When the temperature reached 95 ^O^C, 4 ml of 38.8 mM trisodium citrate was added to the solution. After few minutes, the color of the mixture turned to ruby red. This color change from pale yellow to ruby red, demonstrating the gold nanoparticle formation.

**Green synthesis of GNPs using** ***Hypericum perforatum*** **extract (HPE)**

In this study, the green synthesis method was used for GNPs synthesis. To this, 1 g of HPE was suspended in 4 ml of deionized water and then, vortexed and passed through 0.22 µm filter. The filtrate was mixed with 25 ml of 1 mM HAuCl_4_ and stirred on a hot plate at 37^O^C. The color of solution turned ruby red after 1.5 hr, indicating that HPE stabilized GNPs (HPE-GNP) formation. There was no further color change.

The free gold nanoparticles (Free-GNP) and HPE-stabilized gold nanoparticles (HPE-GNP) were characterized for their shape, size and morphology by UV-visible spectroscopy and atomic force microscopy techniques.

**Animals**

Female pathogen-free C57BL/6 mice, 8-10 weeks old, were obtained from Pasteur Institute of Iran. The animals were housed in 12 hr light/dark cycle with 50±5% relative humidity. All experiments were approved by Animal Ethics Committee of Mashhad University of Medical Sciences (970234).

**EAE induction**

For active induction of EAE, the mice were immunized with subcutaneous injection of MOG amino acid residues 35-55 (MEVGWYRSPFSRVVHLYRNGK) (SBS Genetech CO. Ltd, Beijing, China) emulsified in complete Freund's adjuvant (H37Ra strain; Sigma, St. Louis, MO, USA). Induction was done by a mixture of MOG (300 µg) with complete Freund's adjuvant containing 5 mg/ml heat-killed *Mycobacterium tuberculosis*. 200 µl of the emulsion was injected subcutaneously in the two flanks of each mouse. Each mouse also received 250 ng pertussis toxin (Sigma-Aldrich, St. Louis, MO, USA) intraperitoneally on day 0 and 48 hr later. Animals were weighed and clinical scores were observed blindly by two researchers daily. 

Mice were scored using a 0-7-point scale (Haghmorad et al., 2016 ▶) as depicted in Table 1.

**Experimental design**

Forty-eight C57BL/6 mice were randomly assigned into six groups (8 animals per group). EAE was induced in all mice. Eight animals were grouped to control and received PBS intraperitoneally (i.p). The second group EAE was induced and received GNPs at dose of 1 mM (i.p) and was named Free-GNP group. 

In the other groups HPE-GNP was administered at doses of 50, 150 and 300 mg/kg; These groups were named HPE-GNP1, HPE-GNP2 and HPE-GNP3, respectively. The dose of GNPs in HPE-GNPs was based on the amount of GNPs in the second group (1 mM).

In the last group, HPE in free form was administered at dose of 300 mg/kg.

Treatment began on the day of immunization and continued until termination of the experiment (day 21). On day 21 post-immunization, the mice were sacrificed as per the animal ethics guidelines.

**Histological analysis**

At the end of experiment, mice were transcardially perfused with PBS (phosphate-buffered saline) containing 4% paraformaldehyde and then, sacrificed. Spinal cords were removed and fixed overnight and embedded in paraffin. 5-μm paraffin-embedded sections were dissected, stained with haematoxylin and eosin (H&E), and then, examined by light microscopy to assess leukocyte infiltration. Histological scoring was done by analysis of three fields of slides in a blinded manner (Haghmorad et al., 2016 ▶).

**Evaluation of cytokines**

 For evaluation of cytokines, the splenocytes were isolated and cultured (in triplicate) in 2×10^6^ cells/well in 24-well plates in complete RPMI (Roswell Park Memorial Institute Medium) included 10% FBS (Fetal bovine serum) (Gibco), 100 U penicillin/ml and 100 μg streptomycin/ml (Sigma) in the presence of MOG35-55 (20 μg/ml) and PHA for 72 hr. The culture supernatants were collected and assessed for IL-4 (interleukin-4), IL-6, IL-10, IL-17A, TGF-β (transforming growth factor-β) and IFN-γ (interferon-γ) cytokines using commercial ELISA kits (eBioscience, San Diego, CA) according to the protocol provided by eBioscience.

**Quantitative real-time PCR**

The effects of nanoparticle products on T cell subsets were assessed by analysis of relative expression levels of T cells transcription factors (*T-bet*, *GATA3*, *FoxP3* and *ROR-γt*) using quantitative real-time PCR. Total RNA was isolated from splenocytes by RNA isolation kit (TriPure Isolation Reagent, Roche) and reverse transcribed to cDNA using Prime-Script TMRT reagent kit (Takara Biotechnology, Otsu-Shiga, Japan) according to the kit protocol. mRNA expression was determined by quantitative real-time PCR using Taqman Master Mix (Prime mix EXTaqTM, Takara Biotechnology, Otsu-Shiga, Japan) on the Rotor Gene Q (Qiagen Hilden, Japan).

Primer and probes were synthesized by the Bionner Company (Korea) and Real-time PCR was performed in triplicate and expression normalization was carried out using β2 micro globulin (β2mG) as an endogenous control. The relative expression was calculated based on fold change expression with delta-delta Ct method.

**Statistical analysis**

All data are reported as mean±SEM. A one-way analysis of variance (ANOVA) was conducted for comparison of groups followed by the Tukey *post hoc* test. SPSS19 (IBM SPSS, Armonk, NY) was used for analysis of data. Statistical significance was defined as p<0.05.

## Results

**Characterization of** **GNPs** **products**

**UV-visible spectroscopy**

**Figure 1:**
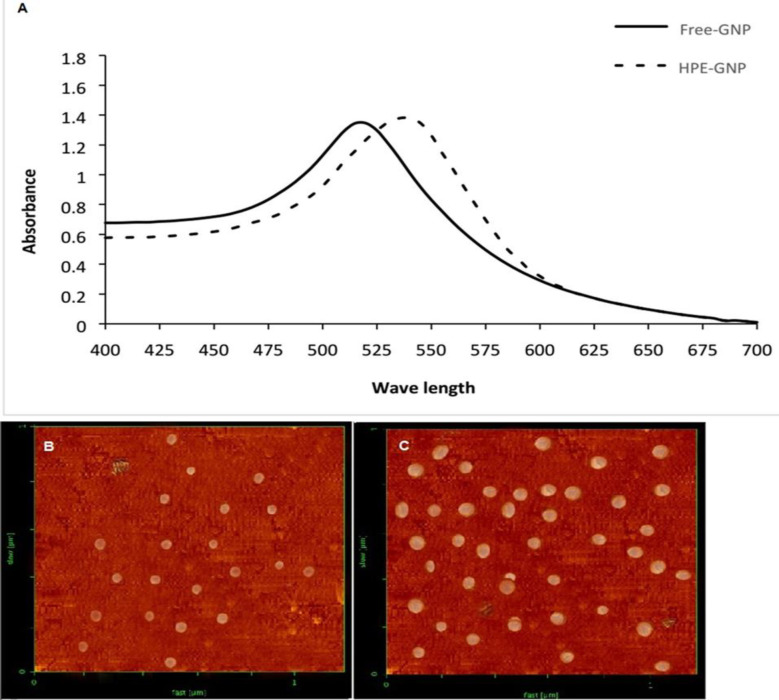
(A) UV-visible spectra of gold nanoparticles upon reduction of HAuCl_4_ by sodium citrate (free GNP, solid line) and after reaction of HAuCl_4_ with HPE (HPE-GNP, dash line). Optical extinction showing a surface plasmon resonance (SPR) band at 517 nm for free-GNP and then shifts to 540 nm due to the effect of HPE on HAuCl_4_. (B) AFM images of gold nanoparticle synthesized using HAuCl_4_ with sodium citrate (free-GNP) and (C) *Hypericum perforatum* extract (HPE-GNP).

The spectral features of gold nanoparticles were assessed by UV-visible spectroscopy. UV-visible analysis of Free-GNPs indicated that they have a peak absorbance at 517 nm (Figure 1A, solid line) corresponding to surface plasmon resonance (SPR) of spherical gold nanoparticles. This property also showed that GNPs were synthesized. UV-visible absorption spectra of HPE-GNP showed a shift to higher wavelength than free-GNPs (540 nm, Figure 1A, dash line). This shift in absorbance intensity is attributed to increased size due to stabilization of GNPs by HPE. This result indicated that the HPE acts as a reducing and capping agent in GNPs synthesis.

**Atomic force microscopy**

The morphology and size of free-GNPs and HPE-GNPs were determined by AFM microscopy. The AFM images showed an average diameter of 20±2 and 35±3.2 nm for Free-GNPs and HPE-GNP, respectively (Figures 1B and C). As seen in the AFM images, all GNPs (except a few particles) were spherical. 

**HPE-GNPs inhibited development of EAE**

**Figure 2:**
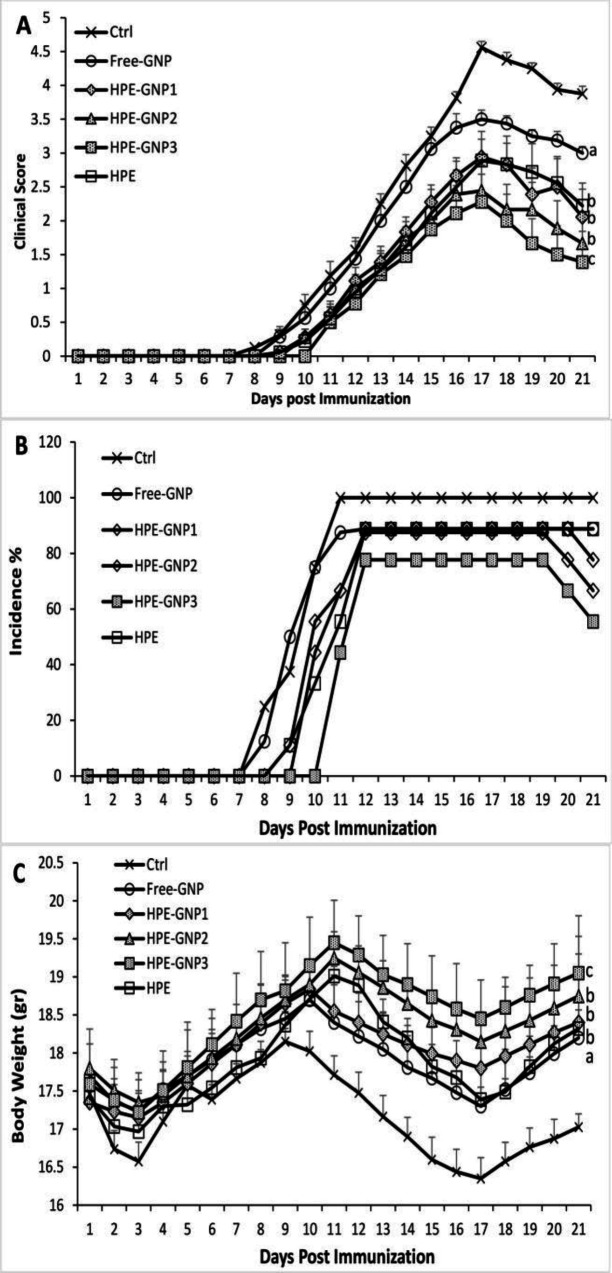
The effect of HPE (*H. perforatum* extract in combination with gold nanoparticles) and HPE (*H. perforatum* extract) treatment on progression of EAE. Clinical assessment of EAE was performed daily and the results are expressed as (A) mean clinical scores (B) incidence of disease and (C) mean body weight. Values shown are mean±SEM. ^a^p<0.05, ^b^p<0.01, and ^c^p<0.001 vs vehicle treated control

Our results showed that HPE-GNPs improved clinical score of EAE. As shown in Figure 2A, all mice in control group developed EAE and the first signs of disease was seen on day 9.6±0.4. Furthermore, maximum mean clinical score (MMCS) reached 4.56±0.11 on day 17. In groups treated with HPE, the MMCS 

decreased to 2.89±0.43 (p<0.01 versus the control) on day 17 and incidence was reduced to 88.8% (Figures 2A and B). 

In mice receiving HPE-GNPs, the clinical score was significantly reduced compared to control, free-GNP and HPE (control 4.56±0.11 vs. HPE-GNP1: 2.94±0.26, HPE-GNP2: 2.44±0.38; p<0.01, HPE-GNP3: 1.83±0.41; p<0.001, HPE: 2.9±0.43; p<0.05) (Figure 2A). 

Furthermore, the disease incidence was reduced in HPE-GNP treatment groups rather than control (Figure 2B). Interestingly, at the end of the study, a decrease in incidence was seen again.

Figure 2C shows that weight loss was considerably improved in the treatment groups, while the improvement was more significant in the groups treated with HPE-GNPs than the Free-GNP/HPE or control (p<0.001). 

Taken together, our results revealed that the treatment with HPE-GNP was associated with more improvement in clinical score and weight loss than free form of HPE or control. 

**Histologic analysis of mice treated by HPE-GNPs**

**Figure 3:**
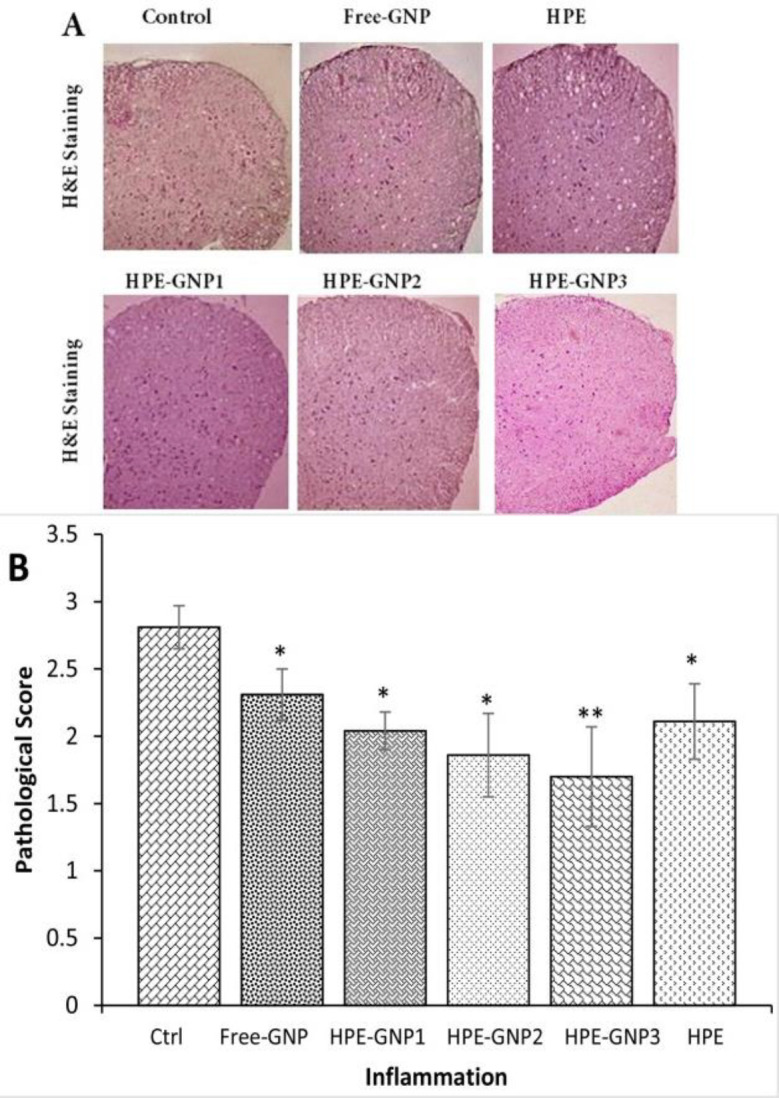
HPE-GNP and HPE treatment reduced CNS inflammation. (A) H&E staining illustrates reduced inflammatory cells into the CNS. (B) Histological results were scored semi quantitatively as described in material section

To determine the effects of HPE-GNPs on histopathology of EAE, the infiltration of inflammatory cells to CNS was evaluated by H&E staining. In untreated control animals, several foci of inflammation were detected due to infiltration of mononuclear cells (Figure 3A). In contrast, in animals treated with free-GNP, inflammation was significantly reduced (Figures 3A and B, p<0.05). In histological analysis of mice treated with HPE-GNPs, only minimal evidence of inflammation was observed (Figures 3A and B). After HPE-GNP3 treatment, a strong decrease of mononuclear cell infiltration was observed compared to the control and HPE-treated group (p<0.01) in the CNS (Figures 3A and B). These data illustrate that the mice were largely protected against EAE.

Values shown are mean±SEM. Statistical analysis was performed by one-way ANOVA. ^*^p<0.05 and ^**^p<0.01, vs vehicle treated control.

**The effects of HPE-GNPs on pro-inflammatory and anti-inflammatory cytokines**

**Figure 4:**
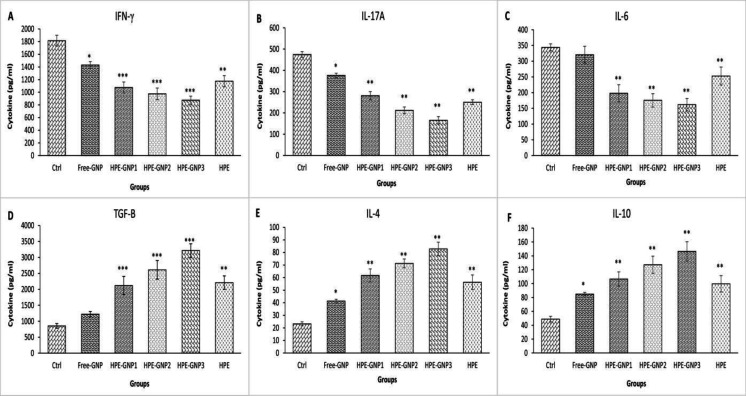
Cytokine pattern produced by spleen cell culture. Mice were immunized and treated as described in material and method. The spleen cells were isolated and cultured in medium with MOG and PHA. Figure shows that HPE-GNP and HPE reduced the level of proinflammatory (A-C) and increased anti-inflammatory cytokines (D-F). Values shown are mean±SEM. Statistical analysis was performed by one-way ANOVA followed by post hoc Tukey's test. ^*^p<0.05, ^**^p<0.01, and ^***^p<0.001 vs vehicle treated control

Cytokine response was evaluated by culture supernatants from splenocytes stimulated with MOG_35-55_. We found that treatment with HPE-GNP1, HPE-GNP2 and HPE-GNP3 significantly decreased IL-6, IFN-γ and IL-17A production (Figure 4, p<0.01 for IL-6 and IL-17A, and p<0.001 for IFN-γ) and significantly increased the level of anti-inflammatory cytokines (IL-4, IL-10 and TGF-β) compared to the control (p<0.01 for IL-4 and IL-10 and p<0.001 for TGF-β).

In group treated with HPE, the level of IFN-γ, IL-17A and IL-6 was decreased (p<0.01), while the level of TGF-β, IL-4 and IL-10 increased compared to vehicle treated mice (Figure 4, p<0.05). Our results also revealed that free-GNP had no effect on TGF-β and IL-6 secretion by splenocytes (p>0.05). Treatment with HPE-GNP3 showed a significant effect on anti-inflammatory cytokines than free-form of drugs or other groups.

**HPE-GNPs inhibit Th1/Th17 and enhance Treg and Th2 responses**

To know the possible role of HPE-GNPs on differentiation of T cells, we assessed the key transcription factors (*T-bet, ROR-γt*, *GATA3* and *FoxP3*) involved in T cell polarization in the brain and spleen of EAE mice.

**Figure 5:**
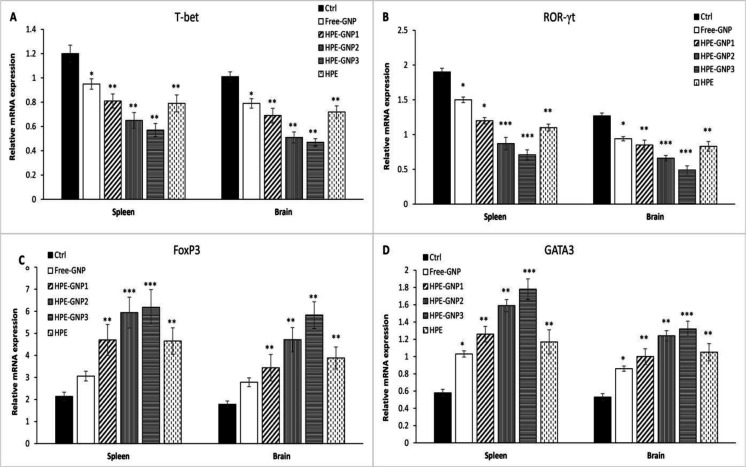
mRNA expression of T cell transcription factors in the spleen and brain of EAE mice. On day 21 after EAE induction, the splenocytes and brain samples were isolated. mRNA expression levels of T cell transcription factors (*T-bet, ROR-γt, GATA3* and *FoxP3*) were assessed by real-time quantitative PCR in the spleen and brain (A-D). Relative expression of genes was analyzed using β2 microglobulin as the internal control

Expression of transcription factors *T-bet* and *ROR-γt* was significantly reduced in the spleen and brain of free-GNP and HPE-treated groups (p<0.05 

and p<0.01, respectively) (Figures 5A and B). An elevated expression of *GATA3*, but not *FoxP3*, was found in free-GNP treated group, while the expression of both transcription factors were elevated in HPE-treated mice (p<0.05) (Figures 5C and D).

The expression of *T-bet* and *ROR-γt* was also significantly diminished by HPE-GNP treatment (Figures 5 A and B). Our results also revealed that HPE-GNPs significantly increased *FoxP3* and *GATA3* expression, in both the spleen and brain (Figures 5 C and D, p<0.01 for HPE-GNP1 and HPE-GNP2 and p<0.001 for HPE-GNP3) compared with the vehicle control.

The results suggested that treatment with HPE-GNPs may contribute to suppressed development of EAE through inhibition of inflammatory T cell (Th1 and Th17) expansion.

Results are expressed as mean±SEM. Statistical analysis was performed by one-way ANOVA followed by post hoc Tukey's test. ^* ^p<0.05, ^**^p<0.01, and ^***^p<0.001 vs vehicle-treated control.

## Discussion

In this research, we used a new method based on GNPs for EAE treatment. Studies have shown that GNPs have low toxicity, simple conjugation potential and they are stable under *in vivo* conditions (Bailly et al., 2019 ▶). Based on the safety of GNPs, as nanocarriers, in human models, it appears that the molecule can be used in preparing human drugs.

Several studies have shown that herbal medicines have the reducing and capping agents that lead to synthesis of biocompatible GNPs (Badeggi et al., 2020 ▶). Therefore, the use of herbal extract, such as *H. perforatum* extract (HPE) allows us to synthesize nontoxic GNPs (HPE-GNP) and make them one of the best nanoparticles for medical applications. 

The results revealed that HPE-GNP not only reduce the clinical and laboratory outcome of EAE, but also has anti-inflammatory effects. Accordingly, the results revealed that HPE-GNP and free-GNP can reduce EAE clinical scores, disease incidence, inflammation (mononuclear cell infiltration) and pro-inflammatory cytokines levels. HPE-GNP also inhibited Th1/Th17 and enhanced Treg and Th2 responses. Therefore, it appears that HPE-GNP potentially have anti-inflammatory effects on the EAE pathogenesis. The results are in parallel with our previous study (Nosratabadi et al., 2016b ▶) which demonstrated that HPE, has anti-inflammatory effects which can reduce clinical and laboratory outcome of EAE. Interestingly, the results demonstrated that HPE-GNP than an equal dose of HPE (300 mg/kg) has higher anti-inflammatory effects in the EAE treatment. The data also demonstrated that HPE-GNP at a low dose has similar effects to the high dose of HPE. 

The molecules also increased the levels of anti-inflammatory cytokines significantly more than HPE and free-GNP. Therefore, it appears that HPE-GNPs use several mechanisms to regulate immune responses in this animal model. 

It is well known that T cells and related cytokines play a crucial role in MS pathogenesis. Previous studies have shown that Th1 cells and their related cytokine (IFN-γ) play critical roles in the EAE/MS pathogenesis (Wagner et al., 2020 ▶). On the other hand, Th2 cells and their related cytokines (IL-4 and IL-10) lead to remission of the disease (Salehipour et al., 2017 ▶). Our results demonstrated that HPE-GNP reduced the level of IFN-γ, while it increased the anti-inflammatory factors (IL-4, IL-10 and *GATA3*). In parallel with our results, several studies showed that treatment with HPE leads to an inhibition in IFN-γ production (Schepetkin et al., 2020 ▶) and an increase in *GATA3* expression (Cabrelle et al., 2008 ▶).

Contrary to the mentioned studies, some researchers have reported that *T-bet* (the Th1 transcription factor) knocked out mice are still susceptible to EAE (Bettelli et al., 2004 ▶). This challenge was resolved by discovery of IL-17-producing CD4+ T cells (Th17 cells) (Capone and Volpe, 2020 ▶). The studies have demonstrated that IL-17 may be more effective than IFN-γ for EAE progression (Komiyama et al., 2006 ▶, Segal, 2019 ▶). Our results demonstrated that HPE-GNP reduced the level of inflammatory cytokines (IL-6, IFN-γ and IL-17A), while it increased the anti-inflammatory cytokines (IL-4, IL-10 and TGF-β). Similar results by Froushani et al. have shown that HPE can decrease IFN-γ and IL-17 and increase IL-10 (Froushani et al., 2015 ▶). Moreover, *in vitro* studies have indicated the anti-inflammatory effect of HPE in mouse hippocampal HT-22 neurons (Bonaterra et al., 2017 ▶). Similarly, a study by Huang et al. also demonstrated the anti-inflammatory effect of HPE in mice infected with H1N1 influenza virus (Huang et al., 2013 ▶). 

IL-10 is another regulatory cytokine in the immune system. Th2 and regulatory T cells are the sources of IL-10 production, but the main source is regulatory T cells (Laidlaw et al., 2015 ▶). Studies have shown that IL-10 has an inhibitory effect in EAE as IL-10 knockout mice are susceptible to EAE (Dai et al., 2012 ▶).

A study on mice infected with influenza A virus showed that HPE could reduce IL-6 and TNF-α and increase IL-10 in infected mice (Xiuying et al., 2012 ▶). Another study also reported that hydro alcoholic extract of HP can decrease IL-17 and increase IL-10 (Froushani et al., 2015 ▶). Taken together, it seems that HPE/HPE-GNPs suppress EAE through inhibitory effects on Th17 and induction of regulatory T cells. 

Histological analysis showed decreased mononuclear cell infiltration in the HPE-GNPs may be a reason for down-regulation of the pro-inflammatory cytokines. Interestingly, the results regarding the expression levels of *T-bet, ROR-γt, GATA3 *and* FoxP3,* as T cell lineage transcription factors, confirmed the hypothesis. HPE-GNPs significantly reduced expression of *T-bet* and *ROR-γt* which are specific for Th1 and Th17 lineage, respectively, and increased *GATA3* and *FoxP3* which are specific for Th2 and CD4^+ ^CD25^+ ^Foxp3^+ ^Tregs lineage, respectively. Reports of the effect of HPE on Th17 and Treg cells are scarce, but a research showed that HPE significantly reduced the IFN-γ production (Froushani et al., 2015 ▶). Another study by Cabrelle et al, showed that hyperforin, a component of HPE, can suppress EAE by decreasing IFN-γ production (Cabrelle et al., 2008 ▶). Additionally, in our previous study, we showed that HPE was able to increase the percentage of Treg cell population in the spleen of EAE mice (Haghmorad et al., 2016 ▶).

These results suggest that HPE-GNPs have neuroprotective effects. In agreement with our results, a study by Oliveira et al. showed that HPE has neuroprotective potential (Oliveira et al., 2016 ▶). Lu et al. reported that HPE plays a neuroprotective role on H_2_O_2_ trauma in PC12 cell line (Lu et al., 2004 ▶). The neuroprotective effect of HPE has also been shown in the neurodegenerative disorders such as depression, and Alzheimer^’^s and Parkinson^’^s disease (Brenn et al., 2014 ▶; Gomez et al., 2013 ▶; Kraus et al., 2007a ▶). Thus, it appears that HPE-GNP not only has immunomodulatory effects, but also has neuroprotective effect.

To the best of our knowledge, this is the first research in which, HPE in nanoform is used for EAE treatment.

This research indicated that HPE in combination with gold nanoparticle was able to inhibit EAE at very low doses compared to HPE alone. This resulted in low toxicity, fewer side effects and enhancement of the therapeutic effect of HPE-GNP. Furthermore, based on the agonistic effects of HPE-GNPs on the immune system in EAE animal in comparison to HPE-treated mice, it may be hypothesized that the nano-herbal drugs can target immune responses and ameliorate MS pathogenesis.

## Conflicts of interest

The authors have declared that there is no conflict of interest.
